# Activation of TRAIL‐DR5 pathway promotes sensorineural degeneration in the inner ear

**DOI:** 10.1111/acel.12437

**Published:** 2016-01-21

**Authors:** Shyan‐Yuan Kao, Vitor Y.R. Soares, Arthur G. Kristiansen, Konstantina M. Stankovic

**Affiliations:** ^1^ Eaton Peabody Laboratories and Department of OtolaryngologyMassachusetts Eye and Ear InfirmaryBostonMAUSA; ^2^ Department of Otology and LaryngologyHarvard Medical SchoolBostonMAUSA; ^3^ Program in Speech and Hearing Bioscience and TechnologyHarvard Medical SchoolBostonMAUSA

**Keywords:** TRAIL, DR5, OPG, cochlea, hair cells, spiral ganglion neuron

## Abstract

Tumor necrosis factor (TNF) family cytokines are important mediators of inflammation. Elevated levels of serum TNF‐α are associated with human sensorineural hearing loss via poorly understood mechanisms. We demonstrate, for the first time, expression of TNF‐related apoptosis‐inducing ligand (TRAIL) and its signaling death receptor 5 (DR5) in the murine inner ear and show that exogenous TRAIL can trigger hair cell and neuronal degeneration, which can be partly prevented with DR5‐blocking antibodies.

## Introduction

The inner ear was previously thought to be deficient in cellular and humoral immunity due to the presence of the blood–labyrinthine barrier established by tight junctions (McCabe, 1989). However, studies over the last decade have shown that inflammatory and immune response in the cochlea play a role in noise‐induced hearing loss and that a variety of inflammatory cytokines are expressed in the cochlea in response to noxious stimuli such as acoustic trauma (Fujioka *et al*., 2014). Among the pro‐inflammatory cytokines, tumor necrosis factor‐alpha (TNF‐α) has been shown to play a role in the loss of cochlear sensory hair cells in animal models (Demirhan *et al*., 2013) and to contribute to sensorineural hearing loss in humans (Svrakic *et al*., 2012). Specifically, exogenous TNF‐α induced loss of hair cells in rat Organ of Corti explants and this TNF‐α‐induced ototoxicity involved the upregulation of a series of apoptosis‐related genes (Dinh *et al*., 2008). Elevated levels of TNF‐α have been detected in inner ears after exposure to noise (Fujioka *et al*., 2006) and ototoxic medications (Park *et al*., 2012). In humans, elevated TNF‐α serum levels have been detected in people with idiopathic sudden sensorineural hearing loss (Demirhan *et al*., 2013) and immune‐medicated sensorineural hearing loss (Svrakic *et al*., 2012).

Our previous work has shown that osteoprotegerin (OPG) – a member of the TNF receptor superfamily – is involved in the regulation of neuronal survival in the inner ear (Kao *et al*., 2013). Loss of OPG expression causes death of spiral ganglion cells and sensorineural hearing loss, in addition to the previously described conductive hearing loss (Zehnder *et al*., 2006). OPG was first discovered as a soluble, neutralizing antagonist that competes with the receptor activator of NF‐κB (RANK) on pre‐osteoclasts and osteoclasts for RANK ligand (RANKL) produced by osteoblasts to inhibit osteoclast formation and function (Khosla, 2001). In addition, OPG was found to interact with another member of the TNF family of cytokines: TNF‐related apoptosis‐inducing ligand (TRAIL). By binding TRAIL, OPG prevents TRAIL from interacting with its receptor and thereby exerts its anti‐apoptosis function (Emery *et al*., 1998). These studies have prompted us to explore physiological and pathological roles of TRAIL in the inner ear.

TRAIL induces apoptosis in a wide variety of cells by binding to a death receptor. In mice, only one death domain‐containing TRAIL receptor, DR5 (mouse KILLER), has been identified (Wu *et al*., 1997). This receptor is a homologue of human DR5 and DR4 (79 and 76% amino acid homology, respectively), and it binds TRAIL with an affinity similar to that of human DR4 and DR5 (Wu *et al*., 1997). TRAIL and TNF‐α have important structural and functional similarities. Specifically, they both contain a TNF domain and form trimeric structures when binding to receptors (Chan, 2007). Both TRAIL and TNF‐α have antitumor activity (Aggarwal *et al*., 1985; Wiley *et al*., 1995) and induce apoptosis (Obeid *et al*., 1993; Degli‐Esposti *et al*., 1997) albeit by different mechanisms (Jin & El‐Deiry, 2006). Both TRAIL and TNF‐α regulate inflammation (Bradley, 2008), at least partly by regulating a pro‐inflammatory transcription factor NF‐kB (Secchiero *et al*., 2003), and both are involved in auto‐immune diseases (Kollias *et al*., 1999; Aktas *et al*., 2005). Due to these similarities between TRAIL and TNF‐α, the importance of TNF‐α for cochlear pathobiology, and our finding of OPG's importance for survival and function of spiral ganglion neurons (Kao *et al*., 2013), we studied the expression and function of TRAIL and DR5 in the inner ear. Using a combination of techniques – including real‐time quantitative RT–PCR, Western blot, *in situ* hybridization, organotypic cell culture, and an auditory cell line – we demonstrate a possible role for TRAIL and DR5 in sensorineural degeneration in the inner ear. Our results suggest a strategy to prevent or treat certain kinds of sensorineural hearing loss.

## Results

### TRAIL and DR5 are expressed in the cochlea

**Figure 1:**
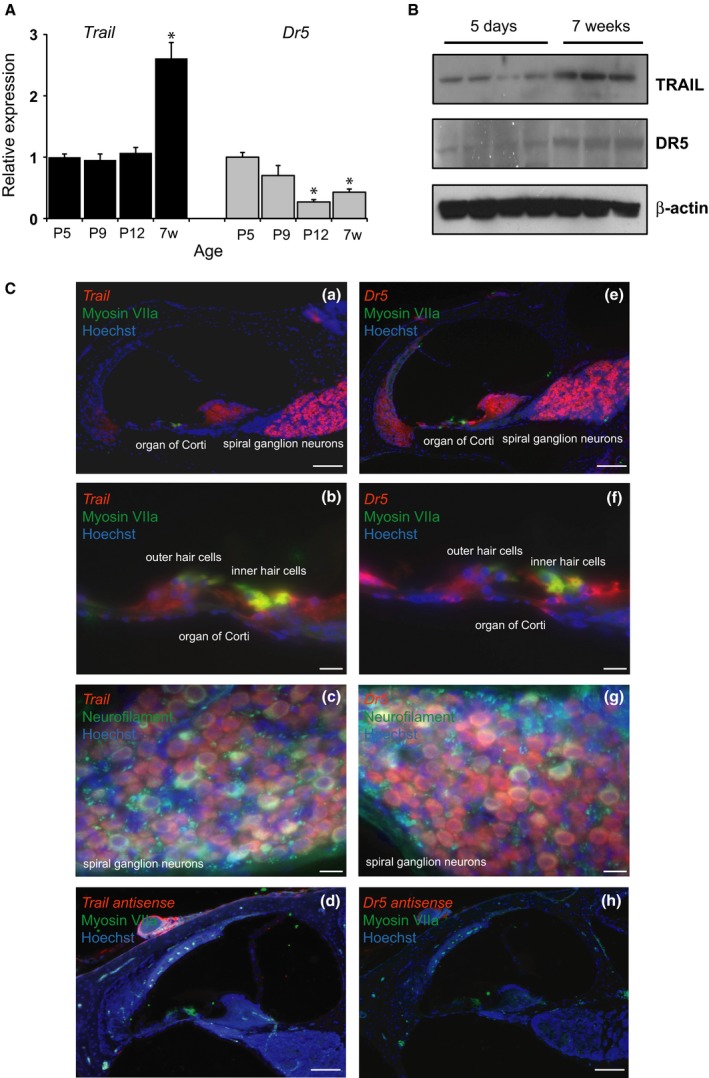
Cochlear expression of *Trail* and *Dr5*. (A) mRNA expression with age, relative to *Trail* mRNA expression at P5. w = weeks. *n* = 5 mice. replicates per age. *P < 0.05. Data are plotted as mean ± SD. (B) TRAIL and DR5 protein expression at 5 days (*n* = 4 mice) and 7 weeks (*n* = 3 mice) of age. (C) *In situ* hybridization for *Trail* (a, b, c), *Dr5* (e, f, g), and antisense controls for *Trail* (d) and *Dr5* (h) in cochlear cross sections. Images of the organ of Corti (b, f) and SGNs (c, g). Scale bar: 100 μm (C(a), (d), (e), (h)) or 20 μm (C(b‐c), (f‐g)). The experiment was repeated in cochlear samples from 3 mice.

To determine whether *Trail* and *Dr5* are expressed in cochlear soft tissues, we used real‐time quantitative PCR (qRT–PCR; Fig. 1A), followed by Western blot (Fig. 1B) and fluorescence *in situ* hybridization to assess cochlear cross sections (Fig. 1C). Expression of *Trail* mRNA was stable in postnatal day (P) 5‐12 cochleae and then increased significantly at 7 weeks. A similar trend was present at the protein level. Expression of *Dr5* mRNA decreased during postnatal development and maturity (Fig. 1A). In contrast, DR5 protein expression increased from P5 to 7 weeks (Fig. 1B), suggesting post‐transcriptional modifications (Fig. 1B). *Trail* and *Dr5* expression localized to specific cochlear cells (Fig. 1C(a) and (e)) in 6‐week‐old mice – primarily hair cells and supporting cells of the organ of Corti (Fig. 1C(b) and (f)) and spiral ganglion neurons (SGNs) (Fig. 1C(c) and (g)). Hair cells and SGNs were identified by concurrent immunohistochemistry for myosin VIIa or neurofilament, respectively. Antisense probes for *Trail* (Fig. 1C(d)) and *Dr5* (Fig. 1C(h)) revealed no nonspecific staining.

### TRAIL treatment causes cellular degeneration in cochlear explants

**Figure 2:**
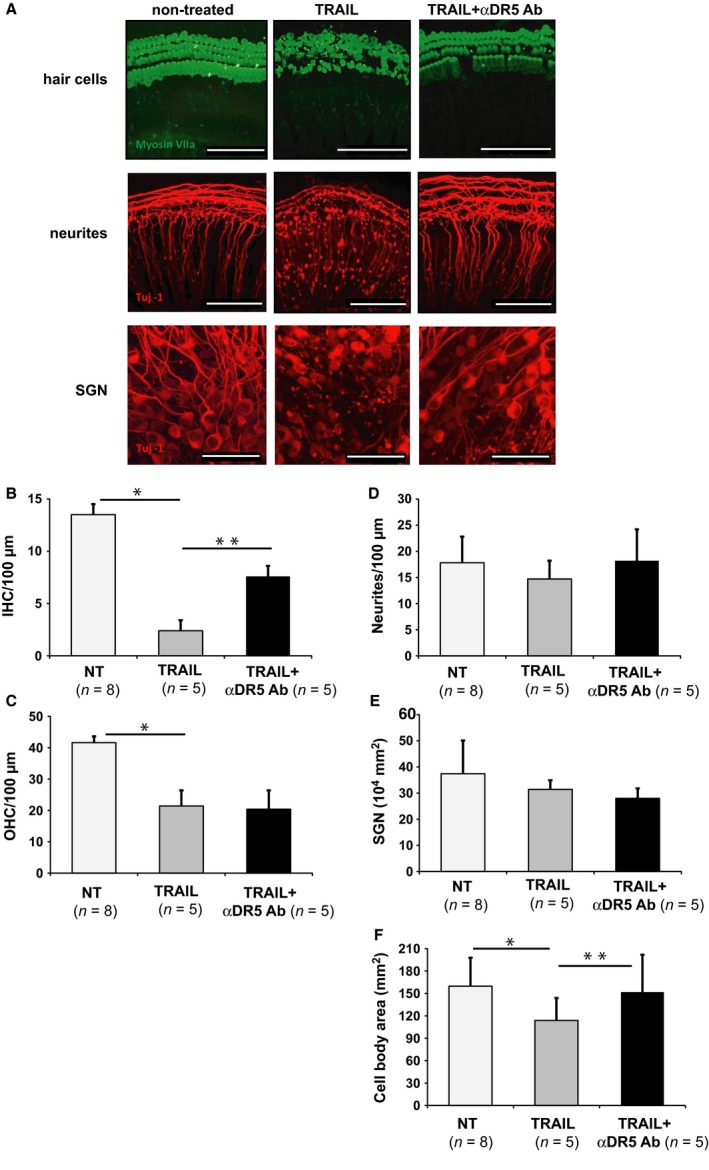
TRAIL treatment damages hair cells and SGNs in cultured murine cochlear explants. (A) Representative images of P4 explants from the same cochlear region that received either 0.1 m PBS (‘nontreated’, NT), 1 μg mL^−1^ TRAIL, or 1 μg/mL TRAIL and 4 μg mL αDR5 Ab. MyoVIIa (green) marks hair cells. Tuj1 (red) marks spiral ganglion neuron (SGN) neurites and somata. Scale bar: 100 μm (top two rows) or 50 μm (bottom row). (B) The number of inner hair cells (IHC) per 100 μm of cochlear length. (C) The number of outer hair cells (OHC) per 100 μm of cochlear length. (D) The number of SGN neurites per 100 μm of cochlear length. (E) The number of SGNs per 10^4^ μm^2^. (F) The distribution of the area of the SGN somata. *P < 0.05, **P < 0.05. *n* = number of different explants. A total of 12 mice were used in these experiments for Figure 2 and Figure S1. Data are plotted as mean ± SD (B–F).

To gain functional insight, cultured cochlear explants were treated with recombinant TRAIL. Representative images are shown in Fig. 2A. Quantification of the results is presented in Fig. 2B–F where ‘*n*’ refers to the number of different animals. TRAIL treatment reduced the number of inner hair cells (IHCs) per 100 μm of cochlear length to 2.4 ± 1.25 (*n* = 5, *P* < 0.05) *re* the control no‐treatment (NT) group (13.5 ± 0.45, *n* = 8). Damage was partially prevented by pretreatment with an anti‐DR5 neutralizing antibody, αDR5 Ab (7.6 ± 1.7, *n* = 5, *P* = 0.041) (Fig. 2A,B). TRAIL treatment also reduced the number of outer hair cells (OHC) per 100 μm to 21.4 ± 5.63 (*n* = 5, *P* = 0.0001) *re* 41.6 ± 1.34 in the control group (*n* = 8), which was not prevented with αDR5 Ab (20.4 ± 6.48; *n* = 5) (Fig. 2A,C). Nonetheless, the morphology of OHCs was greatly improved with DR5 neutralization (Fig. 2A) *re* TRAIL treatment alone.

Although the absolute neurite count per 100 μm did not differ significantly between the groups (Fig. 2A,D), TRAIL caused degenerative neurite beading, which was partly prevented with DR5 neutralization (Fig. 2A). While TRAIL treatment did not result in significant SGN loss, it did cause significant shrinkage of neuronal somata, which could be prevented with αDR5 neutralization. Specifically, the number of neurons per 10^4^ μm^2^ area was 37.4 ± 12.7 in the NT control group (*n* = 9), 31.4 ± 3.5 in the TRAIL‐treated group (*n* = 5), and 28 ± 3.8 in the group treated with anti‐DR5 antibodies and TRAIL (*n* = 5) (Fig. 2E). When quantifying the area of the somata, TRAIL treatment resulted in a smaller area (113.9 ± 35.8 μm^2^) *re* the NT control group (159.7 ± 43.1 μm^2^, *P* = 0.000006), which could be prevented by cotreatment with anti‐DR5 antibodies (151.8 ± 52.7 μm^2^, *P* = 0.0000007) (Figs 2F and S1).

### TRAIL‐induced cell death in cochlear neuroblasts can be prevented by DR5 neutralizing antibodies and OPG

**Figure 3:**
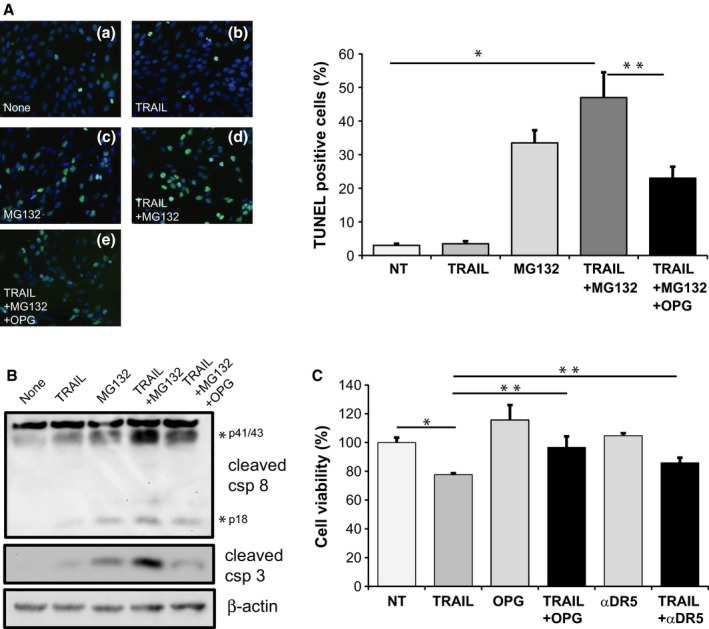
Suppression of TRAIL‐induced cell death in VOT‐33 cells. (A) Detection of apoptosis using TUNEL in cells receiving the following treatment: none (a), 1 μg mL^−1^ TRAIL (b), 10 μm MG132 (c), 1 μg mL^−1^ TRAIL and 10 μm MG132 (d), 1 μg mL^−1^ TRAIL and 10 μm MG132, and pretreatment with 1 μg mL^−1^ OPG (e). (B) Detection of apoptosis using Western blot for cleaved caspase 8 and cleaved caspase 3 in cells receiving the same treatment as in A. (C) Detection of cell viability by MTT assays in cells receiving the following treatment: none, 1 μg mL^−1^ TRAIL, 1 μg mL^−1^ OPG, 1 μg mL^−1^ TRAIL and 1 μg mL^−1^ OPG pretreatment, 4 μg mL^−1^ αDR5, 1 μg mL^−1^ TRAIL, and 4 μg mL^−1^ αDR5. Data are plotted as mean ± SD (A, C). *P < 0.05, **P < 0.05.

As SGN degeneration is typically slow *in vivo* (Kujawa & Liberman, 2009), we studied it in an accelerated model *in vitro*, using a mouse auditory neuroblast cell line, VOT‐33 (Lawoko‐Kerali *et al*., 2004). TRAIL did not induce apoptosis in VOT‐33 cells, as assessed using the TUNEL assays (Fig. 3A(b) compared to no treatment in Fig. 3A(a)). However, treatment with the proteasome inhibitor MG132 – which is known to sensitize tumor cells to TRAIL‐induced apoptosis (Cheong *et al*., 2011; Kahana *et al*., 2011) – caused apoptosis of VOT‐33 cells (Fig. 3A(c)). The cotreatment with TRAIL and MG132 was more effective in inducing apoptosis than MG132 alone (Fig. 3A(d)).

To test whether the TRAIL‐induced death of VOT‐33 cells occurred via activation of the TRAIL‐DR5 pathway, we pretreated VOT‐33 cells with OPG that binds TRAIL and prevents TRAIL from binding DR5 (Emery *et al*., 1998). TRAIL‐MG132 treatment activated caspase 8, a crucial downstream molecule for TRAIL signal transduction (Crowder and El‐Deiry, 2012), as shown by the presence of cleaved caspase 8 in Western blot (Fig. 3B). TRAIL‐MG132 treatment also induced apoptosis, as evidenced by the presence of cleaved caspase 3 (Fig. 3B). Pretreatment with OPG suppressed TRAIL‐MG132‐induced apoptosis in VOT‐33 cells (Fig. 3A(e)) and decreased cleaved caspase 8 and cleaved caspase 3 expression (Fig. 3B), as assessed by Western blot.

When using the MTT cell viability assay, TRAIL treatment reduced VOT‐33 cell viability to 77.65 ± 1.02% *re* the vehicle control (distilled water) (Fig. 3C). This suggests that in addition to promoting cell death, TRAIL may also suppress cell proliferation. Cotreatment with either OPG or αDR5 Ab partially prevented TRAIL‐induced damage and increased cell viability to 96.66 ± 7.65% and 85.92 ± 3.58%, respectively (Fig. 3C).

## Discussion

Our discovery of TRAIL and the death receptor DR5 in the cochlea is novel and may have therapeutic implications. We show that the TRAIL‐DR5 pathway induces degeneration of cochlear sensorineural structures *in vitro*. These results motivate future studies to determine whether inhibition of the TRAIL‐DR5 signaling in the cochlea *in vivo* can prevent sensorineural death and the associated hearing loss. Blocking TRAIL‐DR5 signaling has been shown to be therapeutic in reducing the delayed neuronal damage after transient global cerebral ischemia (Cui *et al*., 2010) and preventing beta‐amyloid neurotoxicity seen in Alzheimer's disease (Uberti *et al*., 2007).

However, full understanding of TRAIL signaling in the cochlea will require future studies of expression of all TRAIL receptors, including those that do not signal apoptosis, because TRAIL function is regulated at the level of receptor expression (Degli‐Esposti *et al*., 1997). In the brain, TRAIL‐DR5 signaling controls not only cell death (Uberti *et al*., 2007; Cui *et al*., 2010), but also inflammation (Hoffmann *et al*., 2009) and neuroproliferation and differentiation (Niu *et al*., 2012).

Several lines of evidence indicate that inflammation plays an important role in sensorineural hearing loss. For example, microbial infections of the middle ear, such as with *Streptococcus pneumoniae* (Dodge *et al*., 1984), *Haemophilus influenzae* type B (Feldman *et al*., 1982), or cytomegalovirus (Bradford *et al*. 2015), can spread to the inner ear and induce inflammation resulting in sensorineural hearing loss. Importantly, such hearing loss can be prevented with anti‐inflammatory medications (Brouwer *et al*., 2013). In addition, tissue damage in inner ear cells, such as due to acoustic trauma, can initiate inflammation and stimulate expression of proinflammatory cytokines, resulting in noise‐induced hearing loss (Fujioka *et al*., 2006). Our study suggests that TRAIL signaling may be involved in sensorineural hearing loss. TRAIL signaling is known to mediate brain injury after inflammation and hypoxia–ischemia (Kichev *et al*., 2014).

In addition to inflammation, autoimmunity is known to play a role in sensorineural hearing loss. Many systemic autoimmune diseases are associated with hearing loss (Bovo *et al*., 2006) while patients with seemingly isolated sensorineural hearing loss can have autoantibodies against inner ear antigens (Greco *et al*., 2011). A comprehensive bioinformatic analysis has revealed that inner ear proteins share sequence similarity with many known immunogenic proteins, which may lead to cross‐reactivity and detrimental immune activation in the inner ear (Platt *et al*., 2014). TRAIL‐DR5 signaling has been implicated in the control of autoimmune diseases in the brain. For example, in experimental autoimmune encephalitis (EAE), TRAIL expression is increased, especially in the activated T cells (Wendling *et al*., 2000). In a similar EAE model, removal of endogenous TRAIL by intracerebral injection of a soluble TRAIL receptor reduced neuronal apoptosis and myelin loss, and prevented neurological disability (Aktas *et al*., 2005). It is likely that, similar to in the brain, TRAIL‐DR5 signaling in the cochlea may mediate autoimmunity, while depending on context and downstream signaling molecules.

Besides DR4 (TRAIL‐R1) and DR5 (TRAIL‐R2), other receptors also bind TRAIL and appear to act as ‘decoys’: DcR1 (TRAIL‐R3), DcR2 (TRAIL‐R4), and OPG (LeBlanc & Ashkenazi, 2003). DcR2 has a truncated nonfunctional death domain, and DcR1 does not contain transmembrane and death domains. Although both receptors are incapable of directly transmitting an apoptotic signal, they may be able to antagonize TRAIL signaling as DcR1 and DcR2 expression is reduced in the postischemic brain, and increased in the protected preconditioned brain. We found that neutralization of DR5 by an anti‐DR5 antibody could only partially rescue TRAIL‐induced apoptosis, and preferentially in IHCs but not OHCs. This partial and cell‐specific effect may be due to putative gradients in cochlear expression of DcR1, DcR2, or an unknown TRAIL receptor. Indeed, IHCs and OHCs are known to express different proteins – for example, prestin is expressed in OHCs only (Zheng *et al*., 2000) while SERPINB6 is expressed in IHCs only (Sirmaci *et al*., 2010). Alternatively, it is possible that the anti‐DR5 antibody could not completely block the function of DR5 due to the complex structure of the multilayered cochlear explants that limited the antibody's access to specific cells. To delineate these possibilities, TRAIL or DR5 deficient mice will be invaluable in future studies *in vivo*.

## Experimental procedures

### Reagents and cells

The anti‐TRAIL antibody (sc‐7877) was obtained from Santa Cruz Biotechnology (Dallas, TX, USA), and the anti‐DR5 antibody (PX064A) was obtained from Cell Sciences (Canton, MA, USA). The anti‐β‐actin (#4970), anticleaved caspase 8 (#8592), and anticleaved caspase 3 (#9662) antibodies were purchased from Cell Signaling (Danvers, MA, USA). Recombinant murine TRAIL/TNFSF10 (1121‐TL‐010) and OPG (459‐MO‐100) were from R & D systems (Minneapolis, MN, USA), and MG132 was from Sigma‐Aldrich (C2211, St. Louis, MO, USA).

Riboprobe combination system‐T3/T7 was from Promega (Madison, WI, USA). *In situ* hybridization solutions were from Roche (Basel, Switzerland), and 1‐step NBT/BCIP Plus suppressor solution was from Thermo Scientific (Cambridge, MA, USA).

The VOT‐33 cell line, a conditionally immortal cell line derived from an embryonic mouse cochlear neuroblast, was a gift provided by Dr. Matthew Holley.

### Mouse strain

Wild‐type C57BL/6J mice were obtained from Jackson Laboratory (Bar Harbor, ME, USA). All animal procedures were approved by the Animal Care and Use Committee of the Massachusetts Eye and Ear Infirmary.

### Fluorescent *in situ* hybridization (FISH) combined with immunohistochemistry

Six‐week‐old C57BL/6J mice were decapitated, and heads were fixed in buffered 4% paraformaldehyde (PFA) after opening the round and oval windows. Cochleae were decalcified in 0.12 m EDTA for 3 days at room temperature, serially dehydrated, embedded in paraffin, and cut in 10 μm sections. After rehydration, cochlear sections were treated with 3% H_2_O_2_ for 20 min to reduce endogenous peroxidase activity, fixed in 4% PFA for 20 min, washed with PBS, digested with proteinase K (10 μg mL^−1^) in PBS for 7 min, and fixed in 4% PFA for 20 min. Sections were immersed in triethanolamine and acetic anhydride solution for 10 min before hybridization. The hybridization mixture, containing the DIG‐labeled antisense or sense probe, was applied to each section and incubated at 42 °C for 16 h. The probes were made from the following nucleotides of the corresponding cDNA sequences: nucleotides 523 to 758 for *Trail* (NM_009425) and nucleotides 275 to 1124 for *Dr5* (NM_020275). All probes were cloned into the pBluescript II SK‐vector. The digoxigenin (DIG)‐labeled single‐stranded antisense and sense RNA probes were prepared using T7 RNA polymerase and T3 RNA polymerase, respectively, with the presence of DIG‐dUTP (digoxigenin DNA labeling mixture (Roche)) according to the manufacture's protocol. Sections were washed at room temperature with 67% 0.2× SSC and 33% TBS (0.1 m TRIS‐HCL, 0.15 m NaCl (pH = 7.5)) for 10 min, 33% 0.2× SSC and 67% TBS for 10 min, and 100% TBS for 10 min, then incubated in a blocking solution (Roche) for 1 h. Sections were incubated with anti‐DIG‐POD antibodies (Roche, 11650300) for 1–2 h, and developed with a TSA PLUS Fluorescence Kit (PerkinElmer, Waltham, MA, USA; NEL744001KT) according to the manufacturer's instructions. After FISH, sections were blocked in 10% normal horse serum for 1 h and incubated with rabbit anti‐Myosin VIIa antibodies (Proteus, Ramona, CA, USA; 25–6790) and chicken antineurofilament antibodies (Millipore, Billerica, MA, USA; AB5539) overnight. Sections were incubated with anti‐rabbit Alexa Fluor 488 antibodies (Jackson Immunoresearch, West Grove, PA, USA; catalog 771‐485‐152) and anti‐chicken Cy5 (Invitrogen, Carlsbad, CA, USA; A21449) for 1 h, followed by nuclear staining with Hoechst. Sections were then mounted with Vectashield (Vector Laboratories, Burlingame, CA, USA) and imaged using an epifluorescent microscope (Axioskop 2 Mot Axiocam; Zeiss, Oberkochen, Germany.).

### Real‐time quantitative RT–PCR

After euthanasia, decapitation and cochlear extraction, cochlear soft tissue was collected by removing the otic capsule through microdissection in RNAlater (Ambion, Austin, TX, USA). Tissue was pooled from both cochleae of a single animal to generate one specimen. Total RNA was purified using RNeasy spin‐columns (Qiagen, Hilden, Germany) according to the manufacturer's protocol and a modification for hypocellular, dense connective tissues. Total RNA was reversely transcribed with Taqman Reverse Transcription Reagents kit (Applied Biosystems, Foster City, CA, USA). Real‐time quantitative RT–PCR was performed using 6‐FAM‐linked fluorescent probes and primers for *Trail* (ID Mm00437174_m1) and *Dr5* (ID Mm00457866_m1) designed and optimized by Applied Biosystems. The measurements were carried out on the Mx3005P machine (Stratagene, San Diego, CA, USA) using 96‐well plates. For each well, the 25 μL reaction contained: 1.25 μL of the 20× probe/primer mix, 1 μL of cDNA template, 12.5 μL of Universal Master Mix (Applied Biosystems, Foster City, CA, USA), and 10.25 μL of distilled water. For each gene, there were 3 technical and 5 biological replicates. Fluorescence data were collected starting with a denaturation step at 95 °C for 10 min, followed by 45 cycles of 95 °C for 15 s and 60 °C for 1 min. Gene expression levels were quantified relative to the 18S rRNA gene and analyzed using the comparative threshold cycle method (Livak & Schmittgen, 2001).

### Western blot

Cochlear soft tissues from two cochleae per mouse were dissected and lysed in RIPA‐DOC buffer (50 mM Tris buffer (pH 7.2), 150 mM NaCl, 1% Triton‐X100, 1% deoxycholate, and 0.1% SDS) with protease inhibitors (Complete, Roche, Basel, Switzerland). Equal amounts of protein extract were loaded per lane, resolved by 4–20% SDS–PAGE, and electro‐transferred onto a PVDF membrane (Immobilon‐P, Millipore, Billerica, MA, USA). Protein detection was performed using the primary antibodies against TRAIL, DR5, cleaved caspase 8, cleaved caspase 3, or β‐actin at 4 °C overnight. After incubation with secondary antibodies for 1 h at room temperature, protein bands were developed using an ECL chemiluminescence detection kit (Pierce, Rockford, IL, USA). Images were quantified using ImageJ (NIH, Bethesda, MD, USA).

### Cochlear explant culture

Four‐day‐old (P4) mice were cryoanesthetized (5 min at 0 °C), decapitated, and disinfected with 70% ethanol (w/v). The skin was removed, and the skull was dissected along the sagittal plane. After removal of brain tissue, each half of the skull was placed in a sterile 60 × 15 mm culture dish (Greiner Bio‐One, Monroe, NC, USA) containing Hanks balanced solution (HBSS) (GIBCO) at 4 °C. Cochleae were isolated from the rest of the temporal bone using a dissecting microscope (Carl Zeiss Microscope, Munich, ALE). The bony labyrinth was removed followed by the spiral ligament and stria vascularis. Cochlear explants containing the organ of Corti and SGNs were cultured in 4‐well 35 × 10 mm culture dishes (Greiner Bio‐One) with a glass coverslip pretreated with BD CellTakTM (BD Biosciences, Franklin Lakes, NJ, USA) to facilitate tissue attachment on the surface of coverslips. We focused on culturing the middle part of the cochlea, consisting of the upper basal and lower apical turn of the cochlea, because its integrity was most robust after dissection and culture. The culture medium was DMEM (Invitrogen) containing 1% ampicillin solution (GIBCO) and 1% GlutaMAX^™^ (Invitrogen). To inhibit the effect of CellTak^™^, the culture medium was not supplemented with 10% FBS 1× (Sigma‐Aldrich) in the first 24 h. The culture plate was incubated at 37 °C in 5% CO_2_ for 24 h until the beginning of the experiment. The explants were treated with (1) 1 μg mL^−1^ TRAIL, or (2) 4 μg mL^−1^ anti‐DR5 antibody pretreatment for 3 h followed by cotreatment with 4 μg mL^−1^ anti‐DR5 antibody and 1 μg mL^−1^ TRAIL, or (3) 20 μL of 0.1 m PBS as a negative control.

### Immunohistochemistry and confocal microscopy

After 48 h of treatment, the specimens were washed twice in 0.1 m PBS solution, fixed with 4% paraformaldehyde for 20 min, permeabilized for 30 min in 0.1 m PBS containing 1% Triton X‐100 (1%) and 5% normal horse serum (NHS), and incubated with primary antibodies overnight – rabbit polyclonal antimyosin VIIa (Proteus Biosciences Inc., Ramona, CA, USA) and mouse monoclonal anti‐Neuronal Class III β‐Tubulin antibody (Covance Research, Dedham, MA, USA). Specimens were washed three times in 0.1 m PBS and stained with the secondary antibodies – anti‐mouse Cy3‐red (Jackson Immuno Research) and anti‐rabbit Cy 2‐green (Jackson Immuno Research, West Grove, PA, USA) for 80 min. Specimens were washed twice with 0.1 m PBS, mounted in Vectashield^®^ solution, and inspected using confocal microscopy (Leica SP5 Confocal, Wetzlar, Germany) with cuts of 0.5 micrometers per slide. The samples were evaluated using 20×, 63×, and 126× magnification. For representative documentation of the morphology of each specimen, the photographs were taken from the central region while stepping in Z in 0.5 μm‐steps through the entire thickness of the specimen. All slices were merged to reconstruct the full thickness of the specimen in a single image using Leica software. The counting of inner and outer hair cells and nerve fibers was performed over 100 μm distance. The number of neurons and the area of their somata were quantified in an area of 10^4^ μm^2^ using ImageJ.

### MTT assay

Cultured VOT‐33 cells were treated with 1 μg mL^−1^ recombinant TRAIL overnight. This concentration of TRAIL was chosen after treating VOT‐33 with different concentrations of TRAIL ranging from 10 ng ml^−1^ to 1 μg mL^−1^, according to published reports (e.g., MacFarlane *et al*., 2000). As VOT‐33 cells were relative resistant to TRAIL, the concentration of 1 μg mL^−1^ was selected. Ten microlitres of 12 mm MTT (Invitrogen) was added in each well to detect cell viability. The optical density (O.D.) at 540 nm of each well was measured using the SmartSpect^™^ Plus spectrophotometer (Bio‐Rad, Hercules, CA, USA). The average O.D. value of the VOT‐33 cells treated with PBS (NT) was set as 100% and used to normalize O.D. values of each treatment. To prevent TRAIL‐induced cell death, the cells were pretreated with either 1 μg mL^−1^ recombinant OPG or 4 μg mL^−1^ α‐DR5 neutralizing antibodies.

### TUNEL assay

VOT‐33 cells grown on coverslips were first pretreated or not treated with 1 μg mL^−1^ recombinant OPG for 1 h and then were treated overnight with 1 μg mL^−1^ recombinant TRAIL, 10 μm MG132, both TRAIL and MG132, or DMSO (NT) in the presence or absence of OPG. Cells were fixed with 4% paraformaldehyde, and the TUNEL assay was performed using the DeadEnd^™^ fluorometric TUNEL system (Promega) according to the manufacturer's instructions. Cell nuclei were marked using Hoechst stain. The results were observed through epifluorescent microscopy (Axioskop 2 Mot Axiocam; Zeiss). The percentage of TUNEL positive cells (green fluorescence) was counted relative to the total number of cells.

### Statistical analysis

Windows Excel 2013 was used for statistical analysis. The *t*‐test was used to analyze quantitative variables. A *P* value <0.05 was considered significant. Data are expressed as mean ± standard deviation (SD).

## Author contributions

S‐Y.K., V.Y.R.S., and K.M.S designed research. S‐Y.K, V.Y.R.S., A.G.K., and K.M.S. performed experiments. S‐Y.K., V.Y.R.S., and K.M.S. analyzed data. S‐Y.K. and K.M.S. wrote the manuscript.

## Conflict of interest

The authors declare that they have no conflict of interest.

## Funding

This study was supported by grants from the Department of Defense grant W81XWH‐15‐1‐0472, the Bertarelli Foundation, the Nancy Sayles Day Foundation, and the Lauer Tinnitus Research Center (all to K.M.S.).

## Supporting information


**Fig. S1.** Distribution of the area of somata of SGNs. NT: SGNs treated with dH_2_O; TRAIL: SGN treated with 1 μg mL^−1^ TRAIL; αDR5 + TRAIL: SGNs pretreated with 4 μg mL^−1^ αDR5 Ab followed by 1 μg mL^−1^ TRAIL treatment.
